# Epilepsy from Pressure upon the Brain

**Published:** 1849-11

**Authors:** James A. Meigs

**Affiliations:** Student of Medicine


*Epilepsy from pressure upon the Brain.* (*Clinic of Jefferson Medical College.*) Reported by Mr. James A. Meigs, Student of Medicine.

A case of considerable importance in surgery, was presented by Professor Pancoast to the class of Jefferson Medical College, on Saturday, January 13th, 1849.

The patient, a lad aged 14 years, had, about nine years previous, received a severe blow upon the sinciput, just over the left orbital ridge, by being precipitated from a cart upon a pile of stones. He was taken up insensible, but under judicious treatment recovered, and was to all appearance perfectly well. Some time after, when the circumstance was almost forgotten, the patient was suddenly seized with epileptic fits, a disease with which, prior to the accident, he had never been troubled. These untoward symptoms gradually increased in frequency and violence, until it was not uncommon for them to recur ten and even twenty times *per diem.*

Coincidently with this epileptiform condition, a slow but progressive decay of his mental faculties became evident, till it was finally feared a total alienation of his mind might supervene. As indicative of this, his features were impressed with the peculiar fatuous expression of confirmed epileptics, while his whole conduct evinced a moody and abstracted state of mind.

These abnormal symptoms had, thus far, been steadily increasing in magnitude and violence, despite the various and well directed remedies employed, when the patient was placed under the charge of Professor Pancoast, who, after a careful investigation, both of the history of the case and the condition of the lad, became convinced that the evil resulted from the pressure of a portion of the vitreous table of the os frontis, upon the anterior lobe of the left cerebral hemisphere. This projection of the bone he thought had been undoubtedly established at the time the accident occurred, but had not manifested itself by its alarming results—the child being then very young—until the brain became considerably developed.

Here then was an extremely delicate point for the formation of a diagnosis, and the establishment of the consequent treatment. The question forcibly presented itself, whether to operate or not.

If the meninges of the brain were inflamed, or the orbital plate broken, it was obvious that no benefit would accrue, and the patient be needlessly subjected to a painful operation. Again, if the frontal sinuses existed to any extent, the danger was manifest of forming an aerial fistula, which would be extremely difficult, if not impossible, to cure.

Notwithstanding these manifold obstacles, the operation was resolved upon, inasmuch as it seemed to give the lad the only chance for his recovery.

His father assenting, the lad was brought before the class on the 17th of January. He was placed upon a table in the clinical room, and as a return of his paroxysms during the operation was feared, he was held firmly down by several assistants. A sort of triangular opening was made, the flaps of which being turned back, the pericranium was exposed. This was divided, and the branches of the supra-orbital and frontal arteries, the hemorrhage from which was considerable, were taken up. The trephine was now applied immediately above the superciliary ridge, and as near the depression as possible. Extreme caution was necessary at this point of the operation, this being a difficult and dangerous place for the application of the trephine. In this case the danger was increased by the incessant struggling and resistance on the part of the patient. A circular piece of the skull was removed, having upon its inner face a spiculum of bone pressing upon the dura mater, thus triumphantly verifying the diagnosis. The dura mater was perfectly healthy, presenting its usual opaque pearly hue.

The edges of the periosteum being brought together, and the flaps laid down and supported by a compress of wetted lint, lightly held in its place by adhesive strips, the patient was transferred to one of the clinical wards of the institution. Here he remained during the ensuing month, under the attendance of Drs. Rand and Horner.

For some time after the operation, it was frequently noticed as a fact worthy of consideration, that any attempts to approximate the lips of the aperture closely, and thereby dispose them to heal at once, were speedily followed by a return of the epileptic paroxysms, which were as readily dissipated by the immediate removal of the approximating force. The same disagreeable results were also found to be induced by the slightest indulgence in any highly nutritious or stimulating aliment.

In addition, therefore, to cold applications to the head, absence of light, and the scrupulous avoidance of all anodyne preparations, which were resorted to immediately after the operation, the lad was kept upon a spare diet, and the aperture allowed to remain open for nearly a month. The judicious nature of this treatment was soon made manifest, by the happy restoration of the lad to mental and physical health.

The scar necessarily left by the operation is scarcely perceptible, while the aperture is filled up with a cartilaginous deposit, as is evident from the resistance offered upon pressure.

The lad is now (August, 1849) employed by his mother to run errands, and attend occasionally to a little store which she keeps in this city. In his daily conduct he evinces an intelligence and physical strength usual to lads of his age and condition in life.

